# Nomophobia in the association between sport anxiety and happiness: Evidence from a cross-sectional mediation model

**DOI:** 10.1371/journal.pone.0358857

**Published:** 2026-10-14

**Authors:** Mehmet Metin

**Affiliations:** Cukurova University, Faculty of Faculty of Sport Sciences, Adana, Turkiye; Universidad Santiago de Cali, COLOMBIA

## Abstract

This study examines the relationship between sport anxiety and happiness by focusing on the mediating role of nomophobia among university students. Grounded in the Transactional Model of Stress and Coping and Uses and Gratifications Theory, the study uses these theoretical perspectives only as conceptual frameworks for interpreting the observed statistical associations. The sample consists of 390 undergraduate students (48.2% female, 51.8% male; all participants were aged 18 years or older) enrolled in Faculties of Sport Sciences. Data were collected using the Sport Anxiety Scale-2 (SAS-2), the Nomophobia Scale (NMP-Q), and the Happiness Scale. Mediation analysis was conducted using Hayes PROCESS macro (Model 4) with 5,000 bootstrap samples to test indirect effects. The findings indicate that sport anxiety is significantly negatively associated with happiness while being positively associated with levels of nomophobia. In contrast, nomophobia shows a significant positive association with happiness. The mediation results reveal a significant indirect effect, indicating a competitive mediation pattern. Specifically, while sport anxiety is directly associated with lower levels of happiness, it is also indirectly associated with higher levels of happiness through higher nomophobia scores. These findings suggest that the observed statistical pattern may be compatible with a compensatory interpretation of nomophobia. However, because of the cross-sectional design, this interpretation should be regarded as a possible explanation rather than evidence of an underlying psychological process. The study contributes to the literature by highlighting the complexity of the statistical associations among sport anxiety, nomophobia, and happiness.

## Introduction

The rapid expansion of digital technologies has fundamentally reshaped everyday life, influencing communication, education, work, and psychological functioning. Among these technologies, smartphones have become indispensable tools that facilitate information access, social interaction, entertainment, and the management of daily activities. While these technologies provide numerous benefits, their widespread adoption has also raised concerns regarding problematic patterns of smartphone dependence and their potential implications for mental health and psychological well-being [1,2].

One of the most widely studied phenomena in this context is nomophobia, commonly defined as the fear, anxiety, or discomfort experienced when individuals are unable to access or use their mobile phones [3]. Previous research has consistently associated nomophobia with a range of psychological outcomes, including anxiety, depression, loneliness, stress, problematic smartphone use, and reduced well-being [4–7]. Nevertheless, the existing literature remains inconclusive. Although nomophobia is generally conceptualized as a maladaptive condition, some studies suggest that smartphone-related engagement may also be associated with social connectedness, emotional comfort, or subjective well-being under particular circumstances [8,9]. These inconsistent findings indicate that the psychological significance of nomophobia may be more complex than a simple indicator of problematic technology use.

Previous studies have also suggested that the relationship between problematic smartphone use, internet addiction, and psychological well-being may be reciprocal rather than unidirectional. Higher levels of depressive symptoms have been associated with greater smartphone addiction [10], while lower levels of psychological well-being have been linked to greater problematic internet use [11]. Moreover, motives for internet use have been identified as relevant factors in understanding internet addiction among adults [12]. Taken together, these findings indicate that the relationships among psychological well-being, problematic smartphone use, and internet addiction are complex and may vary depending on individual and contextual factors.

Within sport settings, psychological well-being represents an important component of both athletic performance and academic functioning. Students enrolled in Faculties of Sport Sciences are frequently exposed to competitive demands, performance expectations, academic responsibilities, and physical challenges that may increase psychological stress. One important consequence of these demands is sport anxiety, a multidimensional construct encompassing cognitive worry, somatic anxiety, and concentration disruption experienced before or during sport performance [13]. Previous studies have consistently demonstrated that elevated sport anxiety is associated with less favorable psychological outcomes, including lower subjective well-being, reduced life satisfaction, and decreased happiness [14,15].

At the same time, smartphones have become deeply integrated into the daily lives of university students, including those involved in sport education. Digital technologies now play an important role in communication, learning, information seeking, and social interaction. Consequently, nomophobia has emerged as an increasingly relevant psychological construct among young adults. Although considerable research has independently examined sport anxiety, nomophobia, and happiness, relatively little is known about how these variables are statistically related within a single analytical framework, particularly among sport sciences students.

Several theoretical perspectives provide useful conceptual contexts for understanding these relationships. The Transactional Model of Stress and Coping proposes that individuals respond to stressful situations through various cognitive and behavioral processes aimed at managing perceived demands [16]. Likewise, Uses and Gratifications Theory suggests that individuals actively engage with communication technologies to satisfy different psychological and social needs, including information seeking, social interaction, and entertainment [17]. These theoretical perspectives provide a conceptual framework for interpreting the observed statistical associations among sport anxiety, nomophobia, and happiness. However, because the present study employs a cross-sectional design, these frameworks are used solely to provide conceptual context for interpreting statistical associations rather than to infer causal mechanisms or psychological processes.

The present study addresses this gap by examining the associations among sport anxiety, nomophobia, and happiness in a sample of university students enrolled in Faculties of Sport Sciences. Specifically, we investigated whether nomophobia is statistically associated with the relationship between sport anxiety and happiness using a cross-sectional mediation model. Rather than examining causal pathways, the study aims to clarify the pattern of associations among these variables and to determine whether nomophobia is associated with the indirect relationship between sport anxiety and happiness.

Based on the existing literature and the conceptual framework guiding the present study, the following hypotheses were formulated:

H1. Sport anxiety will be negatively associated with happiness among university students enrolled in Faculties of Sport Sciences.

H2. Sport anxiety will be positively associated with nomophobia.

H3. Nomophobia will be positively associated with happiness after accounting for sport anxiety.

H4. Nomophobia will be statistically associated with the relationship between sport anxiety and happiness in a cross-sectional mediation model.

The conceptual model examined in the present study is presented in Fig 1.

**Fig 1:**
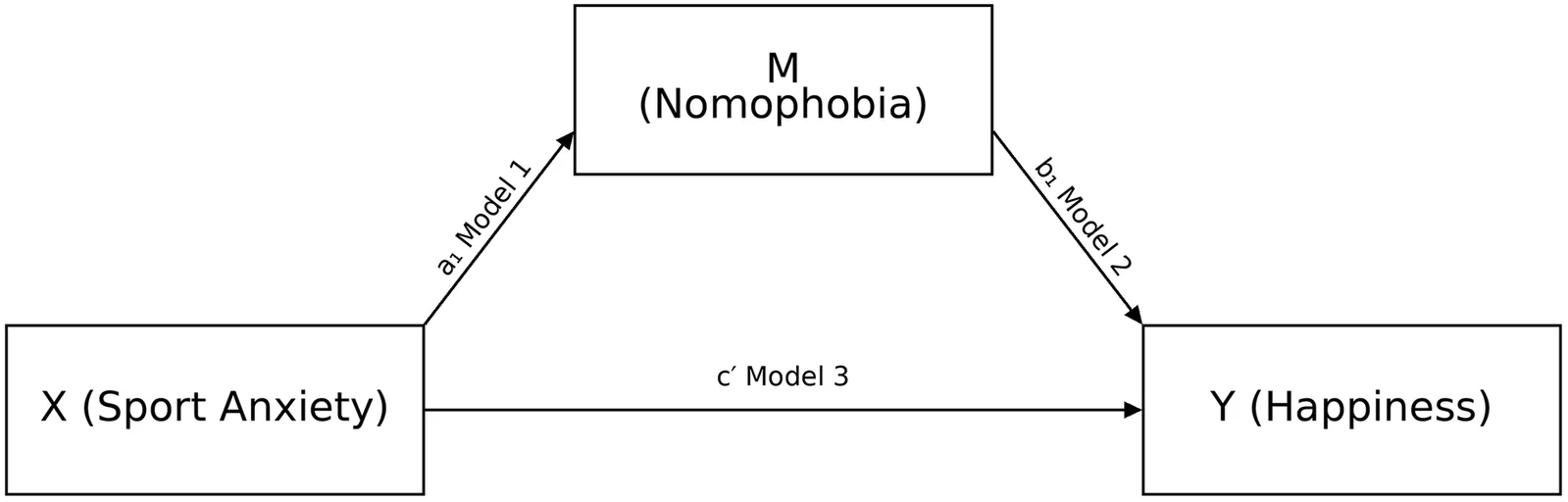
Mediation analysis model.

The figure shows the mediation structure tested within the Hayes PROCESS Model 4 framework. In the model, X represents the independent variable, M represents the mediating variable, and Y represents the dependent variable. Path a represents the association between sport anxiety and nomophobia; path b represents the association between nomophobia and happiness while controlling for sport anxiety; and path c′ represents the direct association between sport anxiety and happiness after accounting for nomophobia. The indirect association between sport anxiety and happiness through nomophobia is represented by the product of paths a × b.

## Research methods and models

The population of the study consists of undergraduate students enrolled in Faculties of Sport Sciences. In the sampling process, criterion sampling, one of the purposive sampling methods, was employed. A total of 413 undergraduate students from the Faculty of Sports Sciences voluntarily participated in the study. Prior to data analysis, all questionnaires were screened for completeness. Twenty-three questionnaires containing incomplete responses were excluded from the analyses. Consequently, the final analytical sample consisted of 390 participants. Data were collected using both face-to-face and online survey administration. Of the final sample, 264 participants (67.7%) completed the questionnaire face-to-face, whereas 126 participants (32.3%) completed it online. Participation was voluntary, and only students aged 18 years or older who provided informed consent were included in the study. Participant recruitment and data collection were conducted between 10/02/2025 and 30/04/2025. The survey forms were administered either face-to-face or online by the researcher. Prior to data collection, participants were informed about the purpose of the study, confidentiality principles, and voluntary participation, and informed consent was obtained.

The study was conducted in accordance with the Helsinki Declaration and approved by the Research Ethics Committee of Çukurova University Faculty of Medicine (Date: 07/02/2025, Approval No: 152). All participants were adults aged 18 years or older. Prior to participation, participants were informed about the purpose of the study, voluntary participation, confidentiality principles, and their right to withdraw from the study at any time without penalty. Informed consent was obtained from all participants before participation. For online participants, consent was obtained electronically before accessing the survey form, while face-to-face participants provided written consent prior to data collection. No personally identifiable information was collected, and no minors were included in the study.

Within the scope of the research, the Personal Information Form, the Nomophobia Scale (NMP-Q), the Sport Anxiety Scale-2 (SAS-2), and the Happiness Scale were used as data collection tools.

The personal information form, developed by the researchers, includes items directly related to the sample group, such as gender, department of study, class level, family income, and whether participants engage in regular physical activity.

The Nomophobia Scale (NMP-Q) [18] was adapted into Turkish [3] and later revised into a five-point Likert format [19]. The scale consists of 20 items across four subdimensions. Items are rated on a five-point Likert scale ranging from 1 (strongly disagree) to 5 (strongly agree), with higher total scores indicating higher levels of nomophobia. The reliability coefficient (Cronbach’s alpha) was reported as 0.92 in the original study. In the present study, Cronbach’s alpha was 0.91.

The Sport Anxiety Scale-2 (SAS-2) [13] was adapted into Turkish [20], who also conducted its validity and reliability analyses. The scale is rated on a 4-point Likert-type format and consists of 15 items across three factors. Items are rated on a four-point Likert scale ranging from 1 (not at all) to 4 (very much), with higher scores indicating higher levels of sport anxiety. The Cronbach’s alpha coefficient for the Turkish version was reported as 0.86. In the current sample, Cronbach’s alpha was 0.91.

The Happiness Scale [21] consists of six items measured on a five-point Likert scale. Items are rated on a five-point Likert scale ranging from 1 (Does Not Describe Me at All) to 5 (Describes Me Completely), with higher scores indicating greater subjective happiness. Cronbach’s alpha internal consistency coefficient was reported as 0.83. Cronbach’s alpha in the present sample was 0.89.

To assess sample precision, Cochran’s sample size formula [22] was used as a reference. According to Cochran’s formula, for large populations, a minimum sample size of 384 participants is recommended assuming a 95% confidence level and a 5% margin of error. After data screening, the final analytical sample consisted of 390 participants, exceeding this reference value. Therefore, the study achieved the targeted level of sample precision under these assumptions. However, because participants were recruited using a convenience sampling method, the findings should not be interpreted as statistically representative of the broader population.

In addition, an a priori statistical power analysis was conducted using G*Power 3.1 [23]. Assuming a medium effect size (f^2^ = .15), α = .05, statistical power = .95, and two predictors, the minimum required sample size was 107 participants. Accordingly, the final sample of 390 participants provided adequate statistical power for the planned analyses.

Mediation analyses were conducted using the PROCESS macro (v4.2) [24] for SPSS. Model 4 was employed to examine the mediating role of nomophobia (M) in the relationship between sport anxiety (X) and happiness (Y). In the analysis, happiness was treated as the dependent variable, sport anxiety as the independent variable, and nomophobia as the mediator. The significance of the indirect effect was tested using a bootstrap method with 5,000 samples, and 95% confidence intervals were reported. Confidence intervals that did not include zero were interpreted as indicating statistically significant indirect effects [24]. In addition, all continuous variables were mean centered prior to analysis to reduce multicollinearity and enhance interpretability [24]. To evaluate the robustness of the findings, an additional adjusted mediation analysis was performed by including gender, department, class, family income, and regular exercise as theoretically relevant covariates. The results of the adjusted model were compared with those of the unadjusted model to examine whether the observed pattern of associations remained stable after controlling for these participant characteristics.

Prior to the mediation analyses, the assumptions underlying linear regression were evaluated. Multicollinearity was assessed using tolerance values, variance inflation factors (VIF), and collinearity diagnostics. Independence of errors was examined using the Durbin–Watson statistic. Standardized residuals, Mahalanobis distance, leverage values, and Cook’s distance were also inspected to identify potential influential observations and assess the adequacy of the regression models.

The results of Harman’s single-factor test are presented in Table 1. Given that the study relied on self-report and cross-sectional data collected from a single source, Harman’s single-factor test was conducted as a preliminary assessment of potential common method variance, following established methodological recommendations [25]. All measurement items were entered into an exploratory factor analysis using an unrotated principal component solution. The results showed that the first factor accounted for 25.25% of the total variance, which is below the commonly referenced threshold of 50%. However, consistent with current methodological recommendations, Harman’s single-factor test alone cannot conclusively determine the presence or absence of common method bias. Therefore, these findings should be interpreted as providing only preliminary evidence that common method variance is unlikely to have substantially influenced the observed relationships.

**Table 1:** Harman’s single-factor test results.

Component	Eigenvalue	% of Variance	Cumulative %
**1**	10.354	25.25	25.25

Note: Extraction Method = Principal Component Analysis (Unrotated Solution)

## Findings

The results regarding the frequency distributions of the demographic and scale questions included in the study are presented in Table 2.

**Table 2:** Frequency distributions of socio-demographic variables.

Variable	Category	Total Nb.	Total %
**Gender**	Female	188	48.2
	Male	202	51.8
**Department**	Physical Education and Sports Teaching	121	31.0
	Coaching	120	30.8
	Sports Management	103	26.4
	Recreation	46	11.8
**Class**	1	74	19.0
	2	93	23.8
	3	141	36.2
	4	82	21.0
**Family Income**	Low	102	26.2
	Medium	257	65.9
	High	31	7.9
**Regular Exercise**	Yes	203	52.1
	No	187	47.9

Table 2 shows that the gender distribution of the participants is relatively balanced, with 48.2% female and 51.8% male. In terms of department, the sample is mainly composed of students from Physical Education and Sports Teaching (31.0%) and Coaching (30.8%), followed by Sports Management (26.4%) and Recreation (11.8%). When examining class level, the majority of participants are in the 3rd year (36.2%), followed by 2nd year (23.8%), 4th year (21.0%) and 1st year students (19.0%), indicating a concentration in upper class levels. Regarding family income, most participants belong to the medium-income group (65.9%), while low-income (26.2%) and high-income (7.9%) groups represent smaller proportions. Finally, in terms of regular exercise, 52.1% of participants report exercising regularly, whereas 47.9% do not. Overall, Table 3 presents the frequency and percentage distributions of the participants’ demographic and lifestyle characteristics.

**Table 3:** Means, standard deviations, skewness, kurtosis, correlations, and reliability values for research variables.

Variables	X̄	SS	1	2	3	Skewness	Kurtosis
**1. Sport Anxiety**	2.09	0.58	(0.91)	0.384**	−0.143**	0.19	−0.30
**2. Nomophobia**	3.02	0.67		(0.91)	0.155**	−0.33	0.32
**3. Happiness**	3.41	0.85			(0.89)	−0.07	−0.67

**p ≤ .01; the values in parentheses are Cronbach’s alpha reliability coefficients.

The findings regarding the mean, standard deviation, Pearson correlation, and Cronbach’s alpha reliability coefficients for the research variables are summarized in Table 3.

The mean scores of the study variables ranged from 2.09 to 3.41. Cronbach’s alpha coefficients ranged from.89 to.91, indicating good internal consistency for all scales. Skewness values ranged from −0.34 to 0.19 and kurtosis values ranged from −0.68 to −0.31, suggesting that the distributions did not substantially deviate from normality. According to the results of the correlation analysis, a positive and moderately significant relationship was found between sport anxiety and nomophobia (r = 0.384; p < 0.01), while a negative and weakly significant relationship was found between sport anxiety and happiness (r = −0.143; p < 0.01), while a positive and low-level significant relationship (r = 0.155; p < 0.01) was found between nomophobia and happiness.

Regression diagnostic analyses indicated that the assumptions underlying the regression models were adequately met. No evidence of problematic multicollinearity was observed (Tolerance = .853–1.000; VIF = 1.000–1.173; Condition Index = 9.03–11.01). Durbin–Watson statistics ranged from 1.46 to 1.58, indicating no substantial violation of the independence of errors assumption. Examination of standardized residuals, Mahalanobis distances, leverage values, and Cook’s distance (maximum = .083) revealed no influential observations that materially affected the regression models.

The independent variable (X) is sports anxiety, the dependent variable (Y) is happiness, and the mediator variable (M) is nomophobia.

Table 4 presents the three regression models together with the decomposition of the total, direct, and indirect associations among sport anxiety, nomophobia, and happiness.

**Table 4:** Cross-sectional mediation analysis of the associations among sport anxiety, nomophobia, and happiness.

Outcome/ model	Predictor/ association	B/ Effect	SE	β	t	p	95% CI
Nomophobia (Model 1)	Sport anxiety	.445	.054	.384	8.19	<.001	[.338,.552]
*Model fit: R^2^ = .147; F(1, 388) = 67.04; p < .001*							
Happiness (Model 2)	Sport anxiety	−.348	.078	−.237	−4.47	<.001	[−.501, −.195]
	Nomophobia	.312	.067	.246	4.64	<.001	[.180,.444]
*Model fit: R^2^ = .072; F(2, 387) = 15.00; p < .001*							
Happiness (Model 3)	Sport anxiety	−.210	.074	−.143	−2.84	.005	[−.355, −.064]
*Model fit: R^2^ = .020; F(1, 388) = 8.06; p = .005*							
**Effect decomposition (PROCESS Model 4)**							
**Effect path**			**Effect**		**BootSE**	**LLCI**	**ULCI**
Total Effect (Sport Anxiety → Happiness)			−0.2095		0.0738	−0.3547	−0.0644
Direct Effect (Sport Anxiety → Happiness)			−0.3483		0.0779	−0.5015	−0.1951
Indirect Effect (Sport Anxiety → Nomophobia → Happiness)			0.1387		0.0336	0.0774	0.2087

*Note.* N = 390. Panel A reports the three regression models. B = unstandardized regression coefficient; β = standardized regression coefficient; SE = standard error; CI = confidence interval. Panel B reproduces the total, direct, and indirect effect estimates from the PROCESS output. Bootstrap estimates are based on 5,000 resamples.

In Model 1, sport anxiety scores were positively associated with nomophobia scores (B = 0.4452, β = .384, p < .001). The model explained 14.73% of the variance in nomophobia and was statistically significant, F(1, 388) = 67.0406, p < .001.

In Model 2, sport anxiety scores were negatively associated with happiness scores when nomophobia was included in the model (B = −0.3483, β = −.237, p < .001). Conversely, after controlling for sport anxiety, nomophobia scores were positively associated with happiness scores (B = 0.3116, β = .246, p < .001). The model explained 7.19% of the variance in happiness and was statistically significant, F(2, 387) = 15.0001, p < .001.

In Model 3, the total association between sport anxiety and happiness was negative and statistically significant (B = −0.2095, β = −.143, p = .005). This model explained 2.03% of the variance in happiness and was statistically significant, F(1, 388) = 8.0563, p = .005.

The effect-decomposition results showed that the total association between sport anxiety and happiness was negative and statistically significant (Effect = −0.2095, SE = 0.0738, 95% CI [−0.3547, −0.0644]). When nomophobia was included in the model, the direct association between sport anxiety and happiness remained negative and statistically significant (Effect = −0.3483, SE = 0.0779, 95% CI [−0.5015, −0.1951]). In contrast, the indirect association through nomophobia was positive and statistically significant (Effect = 0.1387, BootSE = 0.0336, 95% bootstrap CI [0.0774, 0.2087]), as the bootstrap confidence interval did not include zero.

Taken together, the findings indicate that sport anxiety was directly associated with lower happiness, whereas the indirect association through nomophobia operated in the opposite direction. This pattern is consistent with competitive mediation (or statistical suppression), in which the direct and indirect effects have opposite signs. These findings describe the observed statistical associations among the variables and should not be interpreted as evidence of an underlying psychological mechanism or causal process.

To evaluate the robustness of the primary findings, an additional mediation analysis was conducted after adjusting for gender, department, class, family income, and regular exercise. As shown in Table 5, the adjusted model yielded results that were highly consistent with those obtained from the unadjusted analysis. Sport anxiety remained positively associated with nomophobia (B = 0.434, *p* < .001), whereas both the direct effect of sport anxiety on happiness (B = −0.352, *p* < .001) and the indirect effect through nomophobia (Effect = 0.121, BootSE = 0.034, 95% CI [0.060, 0.193]) remained statistically significant. These findings indicate that the observed pattern of statistical associations, including the competitive mediation (suppression) pattern, remained largely unchanged after controlling for the selected demographic covariates.

**Table 5:** Adjusted mediation analysis controlling for demographic covariates.

Path	B	SE	p	LLCI	ULCI
**Sport Anxiety → Nomophobia**	0.434	0.054	<.001	0.327	0.541
**Nomophobia → Happiness**	0.280	0.069	<.001	0.143	0.416
**Direct effect**	−0.352	0.080	<.001	−0.509	−0.195
**Total effect**	−0.231	0.075	.002	−0.379	−0.083
**Indirect effect**	0.121	0.034	—	0.060	0.193

Adjusted for gender, department, class, family income, and regular exercise. Bootstrap confidence intervals were based on 5,000 resamples.

To examine the robustness of the primary findings, an additional sensitivity analysis was conducted by estimating an alternative mediation model in which nomophobia was specified as the independent variable, sport anxiety as the mediator, and happiness as the dependent variable. The alternative model also yielded a significant indirect effect (Effect = −0.115, BootSE = 0.026, 95% CI [−0.170, −0.067]). The direct effect of nomophobia on happiness remained significant (B = 0.312, p < .001), while nomophobia was positively associated with sport anxiety (B = 0.331, p < .001), and sport anxiety was negatively associated with happiness (B = −0.348, p < .001). These findings indicate that the alternative directional specification is also statistically plausible. Accordingly, the cross-sectional design does not permit conclusions regarding the temporal ordering of the variables, and the observed relationships should therefore be interpreted as statistical associations rather than evidence of causal pathways.

This situation points to a more complex relationship pattern, referred to as “competitive mediation” or the “suppression effect,” rather than a classical mediation structure. The findings indicate that sport anxiety was directly and negatively associated with happiness, whereas a positive indirect association with happiness was observed through nomophobia.

## Discussion and conclusion

The findings of the present study generally supported the proposed hypotheses. Specifically, H1 was supported, demonstrating that sport anxiety was negatively associated with happiness. H2 was also supported, indicating that higher sport anxiety was positively associated with higher levels of nomophobia. Consistent with H3, nomophobia remained positively associated with happiness after controlling for sport anxiety. Finally, H4 was supported, as the mediation analysis revealed that nomophobia was statistically associated with the relationship between sport anxiety and happiness through a competitive mediation (suppression) pattern. These findings are discussed in relation to previous literature below.

The findings of the present study confirm that sport anxiety is negatively associated with happiness, which is consistent with prior research in the sport psychology literature [15,26]. These studies have demonstrated that performance pressure, cognitive worry, and somatic anxiety reduce individuals’ subjective well-being and emotional stability. The current results reinforce this established relationship within the context of sport sciences students.

However, the most distinctive contribution of this study lies in demonstrating that nomophobia was statistically associated with the relationship between sport anxiety and happiness through a competitive mediation (suppression) pattern. Rather than indicating a psychological mechanism, this finding highlights the complexity of the observed statistical associations among the study variables.

These theoretical frameworks are presented only as possible conceptual contexts and should not be interpreted as evidence that the observed indirect association reflects these psychological processes. This framework suggests that individuals may employ various cognitive and behavioral responses when encountering stressful situations. Previous research has also reported that digital technologies and online environments are commonly used in relation to stress and anxiety experiences [27,28]. Within this conceptual context, the positive indirect association observed in the present study may be viewed as being compatible with the possibility that smartphone-related engagement accompanies sport anxiety under certain circumstances.

At the same time, the results can be further explained through Uses and Gratifications Theory, which posits that individuals actively use media to fulfill psychological needs such as social interaction, emotional relief, and escapism. In line with this framework, the observed associations may be interpreted as being compatible with the proposition that smartphone-related engagement accompanies experiences of sport anxiety in some individuals. This interpretation is supported by studies indicating that digital engagement can enhance subjective well-being under certain conditions [8,9].

Nevertheless, the positive relationship between nomophobia and happiness identified in this study appears to contradict a substantial body of literature that associates nomophobia with negative psychological outcomes such as reduced well-being and increased anxiety [29]. One possible interpretation of this apparent contradiction is a “short-term coping versus long-term dependency” perspective. Within this interpretation, digital engagement may provide temporary emotional relief for some individuals; however, this possibility was not directly examined in the present study. While digital engagement may provide temporary emotional relief and be associated with increased happiness in the short term, prolonged reliance on such mechanisms may lead to behavioral dependency and adverse psychological consequences over time [30].

This interpretation is further is also compatible with the Behavioral Addiction framework, which conceptualizes nomophobia as a form of behavioral addiction characterized by tolerance, withdrawal, and loss of control [31,32]. Within this context, the findings may be interpreted as indicating that smartphone-related engagement was statistically associated with positive emotional experiences in the present sample. However, because the study employed a cross-sectional design, this association should not be interpreted as evidence of a causal or temporal relationship.

The findings of this study both align with and extend previous empirical research. For instance, Bayıroğlu et al. [33] found that nomophobia and academic achievement can coexist at high levels among sports science students, suggesting that smartphones may serve functional purposes beyond dependency. Similarly, Mavibaş et al. [34] highlighted the role of self-control in moderating the relationship between nomophobia and behavioral outcomes, indicating that individual differences shape how digital engagement influences psychological processes. Furthermore, Antonysamy and Parameswari [35] demonstrated that the effects of nomophobia on well-being are often indirect and mediated by factors such as social media use and exposure to digital content. Together with the present findings, these studies suggest that nomophobia should be conceptualized as a context-dependent and multidimensional construct rather than a unidimensional risk factor.

Importantly, the adjusted mediation analysis yielded results that were highly consistent with those obtained from the unadjusted model. The persistence of the indirect association after controlling for gender, department, class, family income, and regular exercise indicates that the observed pattern of statistical associations remained stable after adjustment for these demographic characteristics. However, because the study employed a cross-sectional design, the findings should be interpreted as statistical associations and should not be considered evidence of causal or temporal relationships.

Alternative explanations should also be considered. For example, the observed pattern may have been influenced by unmeasured third variables, motives for smartphone use, perceived social connectedness, emotion regulation strategies, personality characteristics, or other individual differences. Because these factors were not directly assessed in the present study, their potential contribution to the observed statistical associations cannot be ruled out and should be examined in future longitudinal research.

From a theoretical perspective, this study contributes to the literature in three ways. First, it documents the pattern of statistical associations among sport anxiety, nomophobia, and happiness in a sample of sport sciences students. Second, it highlights that competitive mediation (suppression) may emerge when examining these variables within a cross-sectional mediation framework. Third, it provides a basis for future longitudinal and experimental studies to examine the temporal ordering and robustness of these associations.

Despite these contributions, the relatively low explained variance of the models should be interpreted in light of the complex nature of happiness. Happiness is a multi-determined construct shaped by numerous psychological, social, relational, and environmental factors. Therefore, the modest R^2^ values do not necessarily weaken the theoretical relevance of the model; rather, they indicate that sport anxiety and nomophobia represent only one specific explanatory pathway among many. Future research should incorporate unobserved variables such as personality traits, perceived social support, self-control, resilience, coping styles, and life satisfaction to develop a more comprehensive understanding of happiness in sport-related contexts.

From a practical perspective, the findings suggest that interventions in sports education should not focus solely on reducing digital dependency but should also address the underlying psychological needs that drive such behaviors. Enhancing adaptive coping strategies, emotional regulation skills, and digital awareness may help individuals manage sport anxiety more effectively while minimizing the risks associated with excessive digital reliance.

In conclusion, the present study demonstrates that nomophobia was statistically associated with the relationship between sport anxiety and happiness within a cross-sectional mediation model. The findings revealed a competitive mediation (suppression) pattern in which the direct and indirect associations operated in opposite directions. Because the study employed a cross-sectional design, these results should be interpreted as statistical associations rather than evidence of causal relationships. Future longitudinal and experimental studies are needed to clarify the temporal ordering and robustness of these associations.
